# Supplementary material for: Towards the application of Tc toxins as a universal protein translocation system
Source: Nat Commun. 2019 Nov 20;10:5263. doi: 10.1038/s41467-019-13253-8 (PMC6868009; doi:10.1038/s41467-019-13253-8)
Supplement: Supplementary file 1 — Supplementary Information [file 41467_2019_13253_MOESM1_ESM.pdf]

Supplementary Information

Towards the application of Tc toxins as a universal protein translocation system

Daniel Roderer et al.

Supplementary Figures

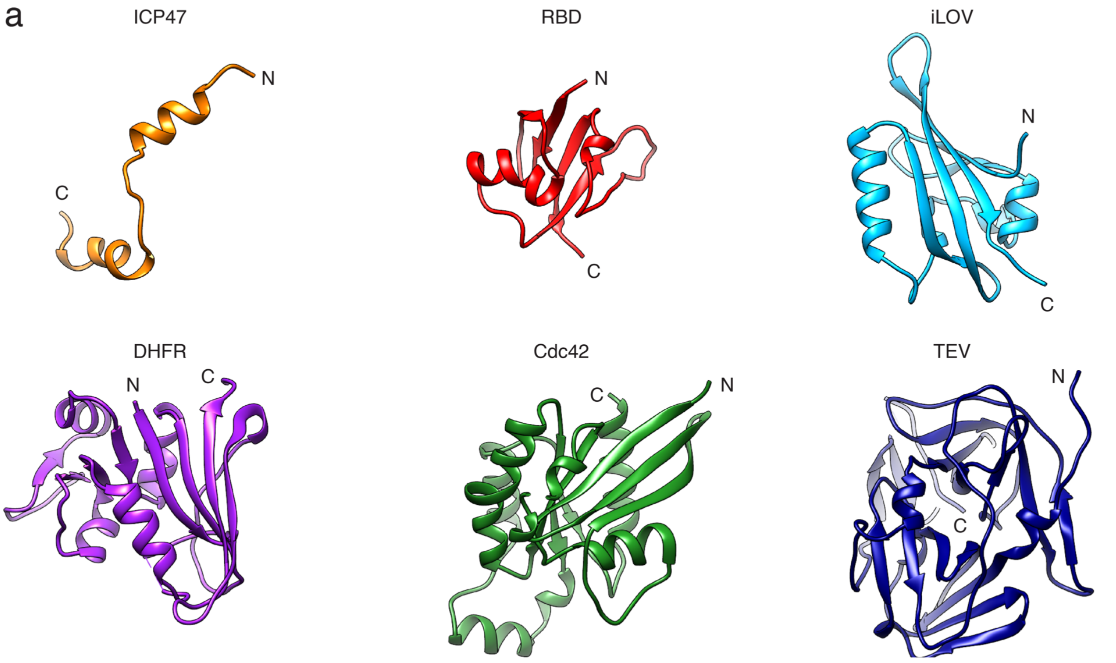

b

```
TccC3HVR     1 MPTIAERIAALKKNKVTDSAPSPANATNVAINIRPPVAPKPSLPKASTSSQPTTH------P-IGAANIKPTTSGSSIVA
TccC5HVR     1 MPEFRTEEAIIKQGSFTGMEEAVYKK------MAKPQTFKRQRAIAAQTEQE-AHESLTNNPSVDISPIKNYTTDSSQIN

TccC3HVR    74 PLSPVGNKSTSEISLPESAQSSSSSTTSTNLQKKSFTLYRADNRSFEEMQSKFPEGFKAWTPLDTKMARQFASIFIGQKD
TccC5HVR    74 A-AIRENRITPAV---ESLDATLSSLQDRQMRVTYRVMTYVDNSTP----------------SPWHSPQEGNSINVGDIV

TccC3HVR   154 TSNLPKETVKNISTWGAKPKLKDLSNYIKYTKDKSTVWVSTAINTEAGGQSSGAPLHKIDMDLYEFAID--------GQK
TccC5HVR   134 SDN----------AYLSTSAHRGFLNFVHKKETSETRYVKMAFLTNAGVNVSAASMYNNAGEEQVFKMDLNDSRKSLAEK

TccC3HVR   226 LNPLPEGRTKNMVPSLLLDTPQIETSSIIALNHGP-------------------VNDAEISFLTTIPLKNVKPHKR
TccC5HVR   204 LKLRVSGPQSGQAEILLPRETQFEV---VSMKHQGRDTYVLLQDINQSAATHRNVRNTYTGNFKSSSAN-------
```

Supplementary Figure 1. Structures of cargo proteins tested for translocation through Tc and sequence alignment of Tcc HVRs. a: Ribbon representation of the structures of the six cargo proteins tested in this study, with N- and C-termini indicated. ICP47: herpes simplex virus protein ICP47 (PDB 1QLO). RBD: Ras binding domain of human CRAF kinase (PDB 1RFA). iLOV: engineered LOV2 domain from *Arabidopsis thaliana* phototropin 2 (PDB 4EES). DHFR: *Escherichia coli* dihydrofolate reductase (PDB 6MT8). Cdc42: human small GTPase Cdc42 (PDB 4YC7). TEV: tobacco etch virus protease (PDB 1Q31). In the case of the NMR structure of the intrinsically disordered protein ICP47, only the N-terminal region (residues 1 – 34 out of 87) has been resolved. In case of Cdc42, one monomer of the homodimer is shown. **b:** Sequence alignment of TccC3HVR and TccC5HVR. Identical and similar residues are highlighted in black and gray, respectively.

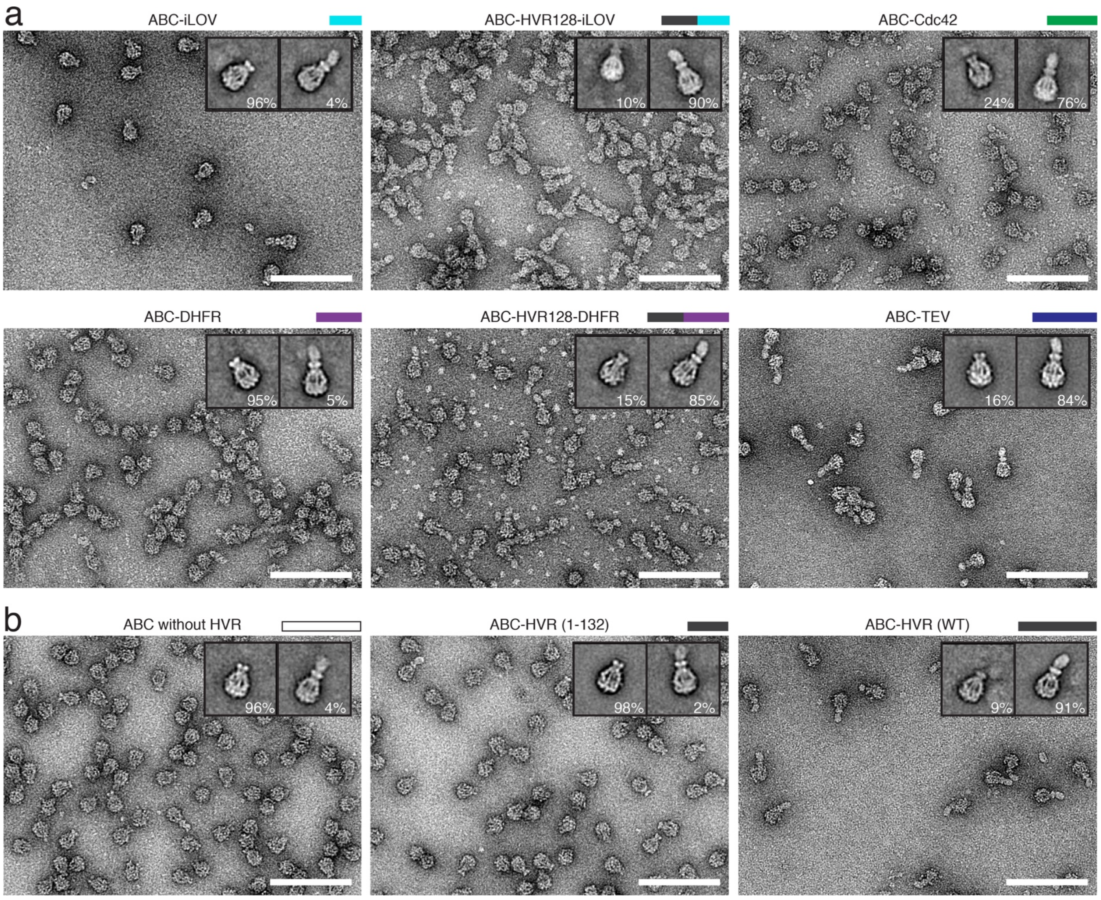

Supplementary Figure 2. Formation of ABC holotoxin with different protein cargos in TcB-TcC. a: Negative stain electron micrographs of ABC after assembly of TcA with TcB-TcC cocoons containing different cargo proteins. Insets: 2D class averages of representative lone TcA pentamer and holotoxin, with the percentages of particles in the class averages shown below. TcB-TcC containing iLOV (13.2 kDa) and DHFR (18.4 kDa) as cargo do not form holotoxins with high affinity (left panels). Fusing the first 128 residues of TccC3HVR to these proteins results in respective cargo sizes of 26.4 and 31.6 kDa, which can efficiently form ABC holotoxin (middle panels). The cargo proteins Cdc42 (20.3 kDa) and TEV (28.1 kDa) form holotoxin with high affinity on their own (right panels). **b:** Negative stain electron micrographs of ABC after assembly of TcA with an empty TcB-TcC cocoon (left), TcB-TcC with a truncated HVR (residues 1 – 132, middle) and ABC(WT) (right). The insets show 2D class averages like described in panel A. The empty cocoon and the cocoon with truncated HVR are inefficient at forming holotoxin. Scale bars: 100 nm.

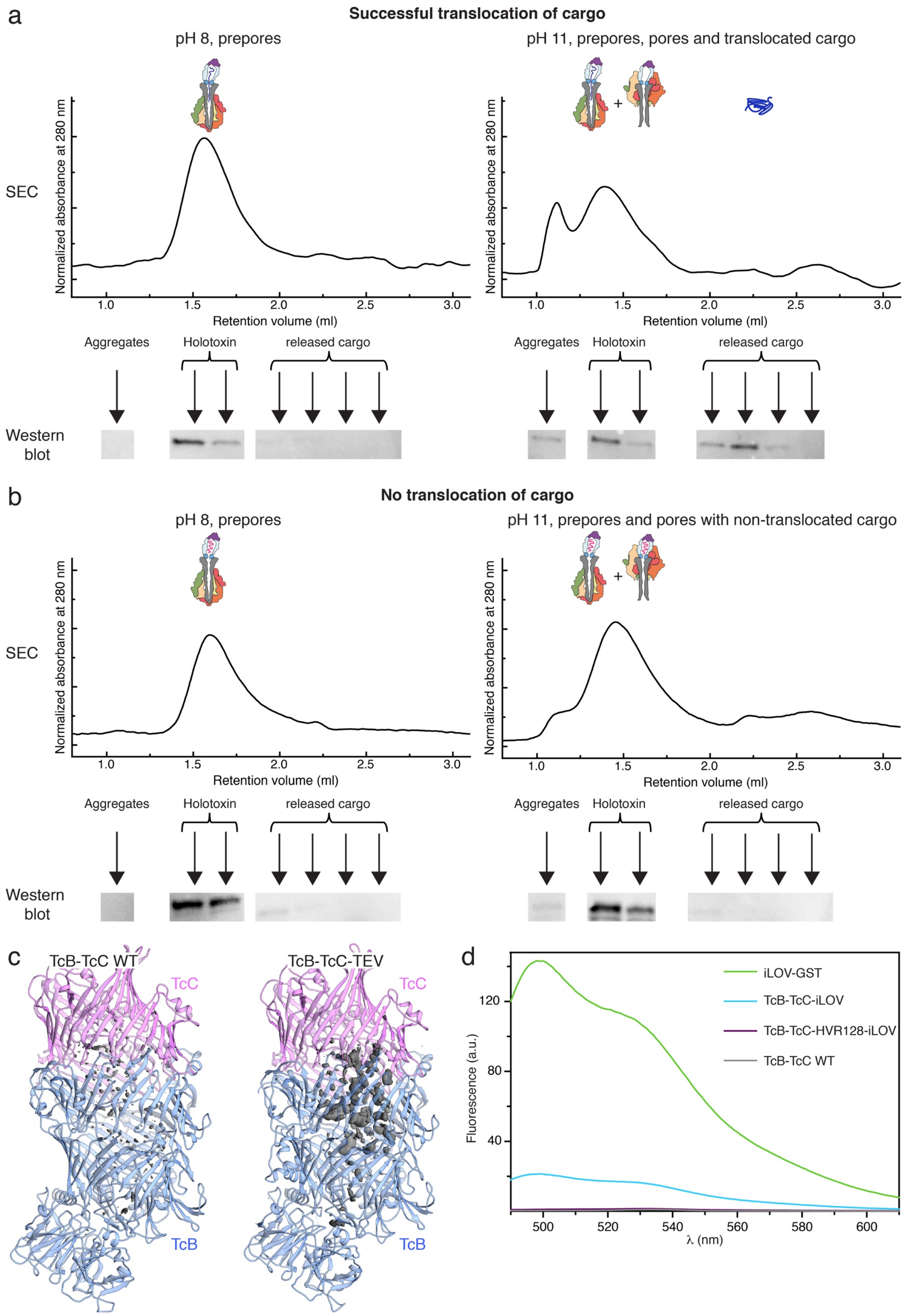

Supplementary Figure 3. Scheme of the *in vitro* translocation experiment of cargo proteins and fluorescence spectroscopy of TcB-TcC-iLOV constructs. a: Successful translocation of the cargo. After incubating the holotoxin for 48 h at pH 8 (as a control where no prepore-to-pore transition occurs) and pH 11 (which induces prepore-to-pore transition), the samples are subjected to SEC on a Superose 6 Increase column. This separates aggregated holotoxin, holotoxin pores and residual prepores (lower retention volumes) from the translocated cargo

(higher retention volumes). Subsequently, SEC fractions are analyzed for the presence of cargo via Western blot using antibodies raised either against the cargo proteins or against TccC3HVR (in case of TccC3HVR fusion constructs). At pH 8, the cargo is still in the cocoon and co-elutes with the holotoxin. At pH 11, the cargo has been released from the holotoxin that transited to the pore state, and therefore also appears at higher retention volumes in comparison to experiments done at pH 8. **b:** No translocation of the cargo. After SEC of samples incubated at pH 11, Western blot analysis shows that the cargo co-elutes exclusively with the holotoxin and cannot be detected at higher retention volumes. **c:** Comparison of the crystal structures of TcB-TcC WT (PDB 4O9X, left) and TcB-TcC-TEV (PDB 6SUQ, right). Electron density inside the cocoon corresponding to the encapsulated HVR and TEV, respectively, is shown in gray and contoured at 0.9 sigma. **d:** Fluorescence emission spectra of 500 nM TcB-TcC-iLOV and TcB-TcC-HVR128-iLOV in comparison with recombinantly expressed and purified iLOV-GST and TcB-TcC(WT). TcB-TcC-HVR128-iLOV is non-fluorescent, while TcB-TcC-iLOV shows ~10% of the fluorescence of iLOV-GST. Thus, the fluorescence produced by TcB-TcC-iLOV indicates that iLOV folds properly and the necessary cofactor FMN is available in the cocoon. The missing fluorescence of TcB-TcC-HVR128-iLOV indicates that the additional 128 residues of the HVR prevent folding of HVR128-iLOV inside the cocoon. Uncropped images of the western blots in panels a and b are provided as a Source Data file.

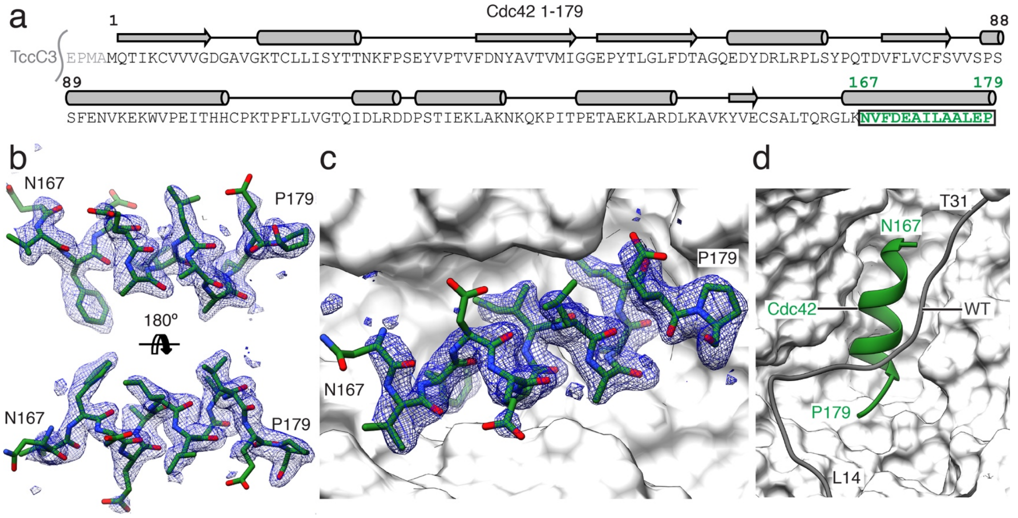

Supplementary Figure 4. Crystallographic density of the Cdc42 C-terminus inside the TcB-TcC cocoon. a: Sequence of the Cdc42 cargo inside TcB-TcC. The first four residues after the TccC3 cleavage site (gray) are an N-terminal extension remaining from restriction cloning. The secondary structure (according to PDB 4YC7) is indicated above the sequence. Residues 167 – 179 at the C-terminus (green) form the α-helix attached to the cocoon's inner surface. **b:** Model of the Cdc42 C-terminus in the crystallographic density map (blue mesh, contoured at 0.92 sigma). Two orientations are shown. **c:** Model of the Cdc42 C-terminus in the crystallographic density map (blue mesh, contoured at 0.95 sigma) within the binding pocket of the cocoon (white surface). **d:** Overlay of the binding pocket of TcB-TcC-Cdc42 including the Cdc42 C-terminus (green) with TcB-TcC(WT). The N-terminus of TcB-TcC(WT) (gray) occupies the space of Cdc42, and the corresponding N-terminal region of TcB-TcC-Cdc42 is not resolved.

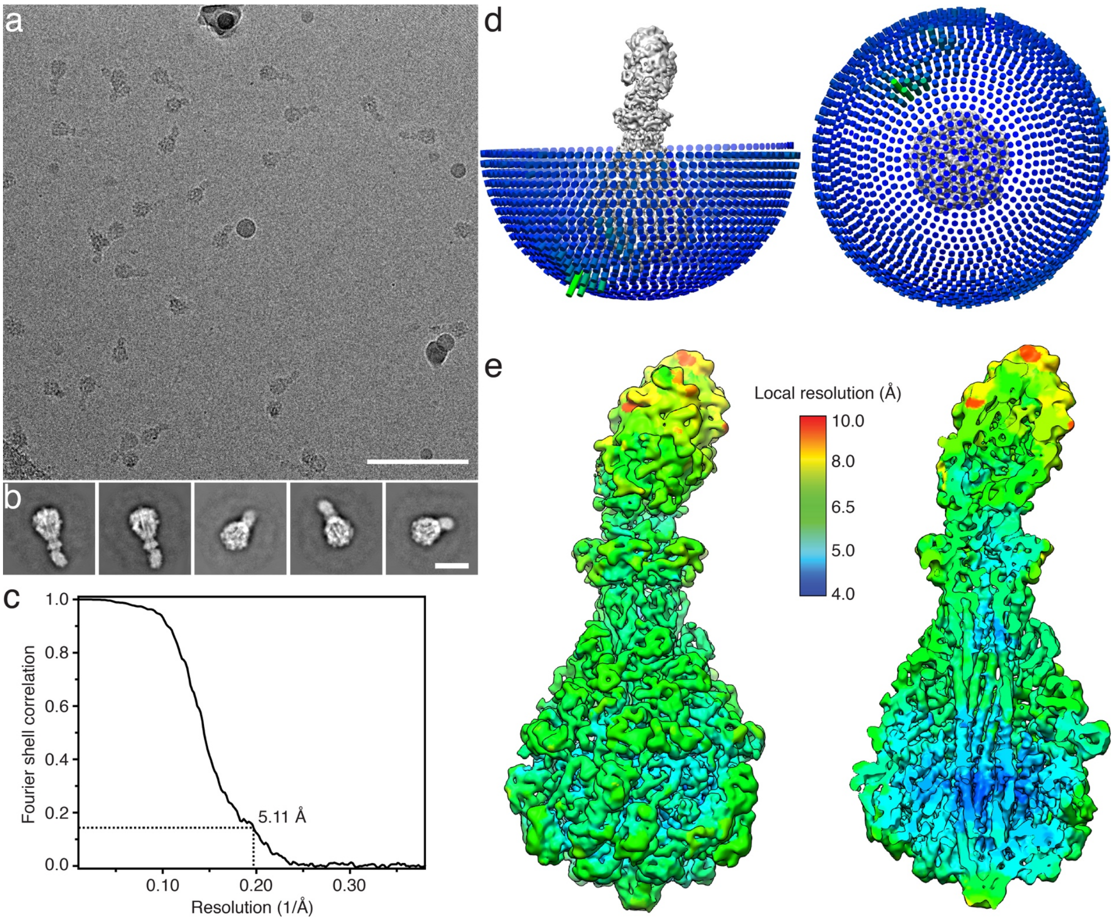

Supplementary Figure 5. Cryo-EM of ABC-Cdc42. a: Representative digital electron micrograph of ABC-Cdc42 at 2.2 µm defocus and a total dose of 65 e^{-} $Å^{-2}$. The image was acquired with a Falcon II direct electron detector on a Cs corrected Titan Krios microscope. Scale bar: 100 nm. **b:** Representative 2D class averages of ABC-Cdc42, showing side views and tilted views. Scale bar: 20 nm. **c:** Fourier shell correlation (FSC) of the obtained cryo-EM map. The dashed line shows the 0.143 FSC cutoff criterion, with a resulting resolution of 5.11 Å. **d:** Side and top view of the angular distribution plot from the final round of 3D refinement. Each cylinder composing the sphere represents a projection view at a particular angle, with length and color proportional to the number of particles at that projection angle. Longer and greener cylinders indicate more particles. **e:** Side view (left) and cross section (right) of the ABC-Cdc42 cryo-EM density map colored according to the local resolution.

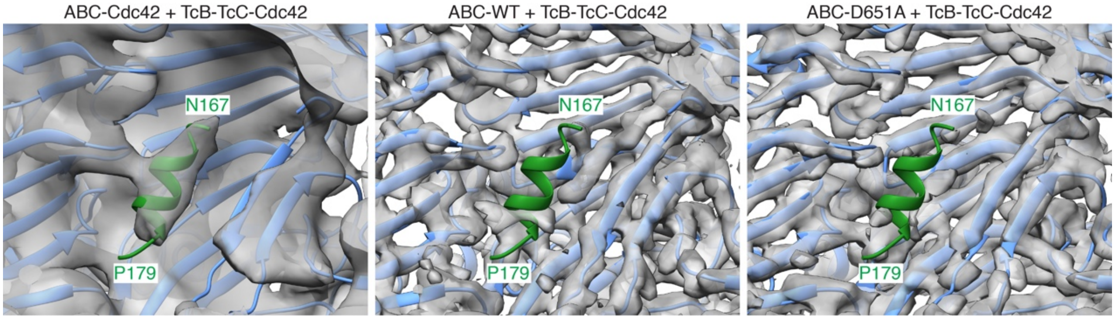

Supplementary Figure 6. Visualization of density corresponding to the α-helix of Cdc42 in different ABC holotoxins. Overlay of the TcB-TcC-Cdc42 model with the cryo-EM maps of ABC-Cdc42 (EMDB 10314, left), ABC(WT) (EMDB 0149, center) and ABC-D651A (EMDB 0150, right). The C-terminal α-helix of Cdc42 is colored green, and the model of TcB is colored blue. Only ABC-Cdc42 shows density corresponding to a folded α-helix inside the cocoon.

Supplementary Tables

Supplementary Table 1. Data collection and refinement statistics (molecular replacement)

	TcB-TcC-TEV	TcB-TcC-Cdc42
**Data collection**		
Space group	$P3_221$	$P2_12_12_1$
Cell dimensions		
a, *b*, *c* (Å)	234.4 234.4 143.2	96.4, 156.6, 179.5
α, β, γ (°)	90, 90, 120	90, 90, 90
Resolution (Å)	47.7-3.7 (3.8-3.7)	48.2-2.0(2.1 -2.0)
R_{merge}	0.5733 (1.626)	0.1855 (2.02)
$CC_{1/2}$	0.972 (0.707)	0.998 (0.688)
$I / \sigma I$	5.3 (2.1)	13.8 (1.5)
Completeness (%)	99.89 (99.96)	99.50 (99.37)
Redundancy	20.5 (21.6)	22.9 (17.8)
**Refinement**		
Resolution (Å)	47.7-3.7 (3.8-3.7)	48.2-2.0(2.1 -2.0)
No. reflections	48,591	18,143
R_{work} / R_{free}	28.5 (26.9)/ 30.7 (30.2)	21.2 (28.7)/ 25.0 (32.0)
No. atoms		
Protein	17064	17004
Ligand/ion		3
Water		1282
B-factors		
Protein	131.02	41.86
Ligand/ion		35.60
Water		42.20
R.m.s. deviations		
Bond lengths (Å)	0.003	0.005
Bond angles (°)	0.55	0.78

Each dataset was collected from a single crystal. Values in parentheses are for highest-resolution shell

Supplementary Table 2. Cryo-EM data processing statistics of ABC-Cdc42.

	ABC-Cdc42 (EMDB-10314)
**Data collection and processing**	
Magnification	59,000
Voltage (kV)	300
Electron exposure (e–/Å^2)	65
Defocus range (μm)	1.0 – 3.2
Pixel size (Å)	1.14
Symmetry imposed	C1
Initial particle images (no.)	99,980
Final particle images (no.)	56,665
Map resolution (Å)	5.1
FSC threshold	0.143
Map resolution range (Å)	4.0 – 10.0

Supplementary Table 3. Overview of the primers that were used for the construction of all plasmids in this study. F, R: forward and reverse primers, respectively.

Mutation	Primer sequence
Insertion of EcoRI restriction site after P680 in TcC, F	CCTTGATCCTGATGGATTAATGCCAGAATTCGCAGAACGCATAGCAGCAC
Insertion of EcoRI restriction site after P680 in TcC, R	GTGCTGCTATGCGTTCTGCGAATTCTGGCATTAATCCATCAGGATCAAGG
Insertion of EcoRI restriction site after K723 in TcC for "HVR45" constructs, F	GCACCAAAACCTAGCTTACCGAAAGAATTCACGAGTAGCCAACCAACCAC
Insertion of EcoRI restriction site after K723 in TcC for "HVR45" constructs, R	GTGGTTGGTTGGCTACTCGTGAATTCTTTCGGTAAGCTAGGTTTTGGTGC
Insertion of EcoRI restriction site after E806 in TcC for "HVR128" constructs, F	CCTTTGAAGAAATGCAAAGTAAATTCCCTGAAGAATTCAAAGCCTGGACT CCTCTAGACAC
Insertion of EcoRI restriction site after E806 in TcC for "HVR128" constructs, R	GTGTCTAGAGGAGTCCAGGCTTTGAATTCTTCAGGGAATTTACTTTGCAT TTCTTCAAAGG
Insertion of NotI restriction site 50 residues upstream of the C-terminus of TcC, F	CATTGATGGACAAAAACTAAATCCACTACGCGGCCGCAGAACTAAAAACA TGGTACCTTCCCTTTTAC
Insertion of NotI restriction site 50 residues upstream of the C-terminus of TcC, R	GTAAAAGGGAAGGTACCATGTTTTTAGTTCTGCGGCCGCGTAGTGGATTT AGTTTTTGTCCATCAATG
Insertion of TccC5HVR after P680 of TcC via EcoRI, F	TATATAGAATTCAGAACAGAAGAAGCGATAATAAAACAGGGTTCCTTTAC GG
Insertion of TccC5HVR directly before the C-terminus of TcC via XhoI, R	TATATACTCGAGTTAGGTACCATTTGCACTGGATGATTTGAAATTACCGG
Insertion of ICP47 after P680, K723 or E806 of TcC via EcoRI, F	GGTTGTGAATTCAGTTGGGCCCTGGAAATGGC
Insertion of ICP47 directly before the C-terminus of TcC via XhoI, R	GGTTGTCTCGAGTTAAGCGTAGTCTGGGACGTCG
Insertion of ICP47 50 residues before the C-terminus of TcC via NotI, R	GGTTGTGCGGCCGCCAGCGTAGTCTGGGACGTCG
Insertion of RBD after K723 or E806 of TcC via EcoRI, F	ATATATAGAATTCAGCAACACTATCCGTGTTTTGTTG
Insertion of RBD before the C-terminus of TcC, XhoI, R	TATATACTCGAGCTACAGGAAATCTACTTGAAGTTCTTCTCC
Insertion of iLOV after P680 or E806 of TcC via EcoRI, F	ATATATACCGGAATTCATAGAGAAGAATTTCG
Insertion of iLOV before the C-terminus of TcC via XhoI, R	TATATATATAGCTCGAGCTTATACATGATCACTTCCATCGAGC
Insertion of DHFR after P680 or E806 of TcC via EcoRI, F	GGTTGTGAATTCATGATCAGTCTGATTGCGGCGTTAG
Insertion of DHFR before the C-terminus of TcC via XhoI, R	GGTTGTCTCGAGTTACCGCCGCTCCAGAATCTCAAAG
Insertion of TEV after P680 of TcC via EcoRI, F	GCAGCGGTCTGGAAGTTCTGTTTCAGGAATTCGGAGAAAGCTTGTTTAAG GGGC
Insertion of TEV before the C-terminus of TcC via XhoI, R	TATACTCGAGTTAGCGACGGCGACGACGATTC
Insertion of Cdc42 after P680 or K723 of TcC via EcoRI, F	ATATATGAATTCATGGCGATGCAGACAATTAAGTGTGTTG
Insertion of Cdc42 directly before the C-terminus of TcC via XhoI, R	ATATATCTCGAGTCAAGGCTCGAGGGCAGCTAGGATAGCCTC
Insertion of Cdc42 50 residues before the C-terminus of TcC via NotI, R	ATATATAGCGGCCGCCCAGGGCAGCTAGGATAGC
Insertion of Cdc42(1-164) 50 residues before the C-terminus of TcC via NotI, R	TATATAGCGGCCGCTCAGACCTCTCTGTGTAAGTGCAGAACAC
Point mutation F680P in TcB-TcC-Cdc42, F	CCTTGATCCTGATGGATTAGAACCCATGGCGATGCAGACAATTAAGTG
Point mutation F680P in TcB-TcC-Cdc42, R	CACTTAATTGTCTGCATCGCCATGGGTTCTAATCCATCAGGATCAAGG
Point mutation W811TAG in TcB-TcC to create HVR(1-132), F	CCCTGAAGGATTTAAAGCCTAGACTCCTCTAGACACTAAGATGGCAAGG
Point mutation W811TAG in TcB-TcC to create HVR(1-132), R	CCTTGCCATCTTAGTGTCTAGAGGAGTCTAGGCTTTAAATCCTTCAGGG
