# Supplementary material for: Towards the application of Tc toxins as a universal protein translocation system
Source: Nat Commun. 2019 Nov 20;10:5263. doi: 10.1038/s41467-019-13253-8 (PMC6868009; doi:10.1038/s41467-019-13253-8)
Supplement: Supplementary file 3 — Reporting Summary [file 41467_2019_13253_MOESM3_ESM.pdf]

nature research

Corresponding author(s): Stefan Raunser

Last updated by author(s): Oct 22, 2019

Reporting Summary

Nature Research wishes to improve the reproducibility of the work that we publish. This form provides structure for consistency and transparency in reporting. For further information on Nature Research policies, see Authors & Referees and the Editorial Policy Checklist .

Statistics

For all statistical analyses, confirm that the following items are present in the figure legend, table legend, main text, or Methods section.

n/a	Confirmed	
☒	☐	The exact sample size (*n*) for each experimental group/condition, given as a discrete number and unit of measurement
☒	☐	A statement on whether measurements were taken from distinct samples or whether the same sample was measured repeatedly
☒	☐	The statistical test(s) used AND whether they are one- or two-sided<br>*Only common tests should be described solely by name; describe more complex techniques in the Methods section.*
☒	☐	A description of all covariates tested
☒	☐	A description of any assumptions or corrections, such as tests of normality and adjustment for multiple comparisons
☒	☐	A full description of the statistical parameters including central tendency (e.g. means) or other basic estimates (e.g. regression coefficient) AND variation (e.g. standard deviation) or associated estimates of uncertainty (e.g. confidence intervals)
☒	☐	For null hypothesis testing, the test statistic (e.g. *F*, *t*, *r*) with confidence intervals, effect sizes, degrees of freedom and *P* value noted<br>*Give P values as exact values whenever suitable.*
☒	☐	For Bayesian analysis, information on the choice of priors and Markov chain Monte Carlo settings
☒	☐	For hierarchical and complex designs, identification of the appropriate level for tests and full reporting of outcomes
☒	☐	Estimates of effect sizes (e.g. Cohen's *d*, Pearson's *r*), indicating how they were calculated

Our web collection on statistics for biologists contains articles on many of the points above.

Software and code

Policy information about availability of computer code

Data collection	Cryo-EM data were collected with EPU version 1.8.
Data analysis	Cryo-EM data were processed using Relion version 1.4 (autopicking, 2D classification) and SPHIRE versions 2017-06-02 and 1.2 (2D classification, 3D refinement, local resolution estimation, local filtering). Movie frames were aligned using MotionCor2.<br>X-ray data were processed and analyzed using XDS and PHENIX version 1.14-3260.<br>Figures of structures were created with Chimera version 1.12.

For manuscripts utilizing custom algorithms or software that are central to the research but not yet described in published literature, software must be made available to editors/reviewers. We strongly encourage code deposition in a community repository (e.g. GitHub). See the Nature Research guidelines for submitting code & software for further information.

Data

Policy information about availability of data

All manuscripts must include a data availability statement. This statement should provide the following information, where applicable:

- Accession codes, unique identifiers, or web links for publicly available datasets
- A list of figures that have associated raw data
- A description of any restrictions on data availability

The cryo-EM map of ABC-Cdc42 has been deposited in the Electron Microscopy Data Bank under accession number 10314. The coordinates of the crystal structures of TcB-TcC-Cdc42 and TcB-TcC-TEV have been deposited in the Protein Data Bank under accession numbers 6SUP and 6SUQ, respectively. The source data underlying Figs 3a-c and Supplementary Figs 3a,b are provided as a Source Data file. Other data are available from the corresponding author upon request.

Field-specific reporting

Please select the one below that is the best fit for your research. If you are not sure, read the appropriate sections before making your selection.

☒ Life sciences ☐ Behavioural & social sciences ☐ Ecological, evolutionary & environmental sciences

For a reference copy of the document with all sections, see nature.com/documents/nr-reporting-summary-flat.pdf

Life sciences study design

All studies must disclose on these points even when the disclosure is negative.

Sample size	Figure 2b,c, SI Figure 2 (2D classification and quantification of TcA and holotoxins): at least 1000 individual particles were subjected to 2D classification and analysis. 2D classes contained 15 - 30 individual particles per class.
Data exclusions	Figure 2b,c, SI Figure 2 (2D classification and quantification of TcA and holotoxins): no 2D classes showing TcA or holotoxin were excluded from the analysis.
Replication	Figure 1c: intoxication experiments were performed in triplicate with qualitatively identical results.
Randomization	n/a
Blinding	n/a

Reporting for specific materials, systems and methods

We require information from authors about some types of materials, experimental systems and methods used in many studies. Here, indicate whether each material, system or method listed is relevant to your study. If you are not sure if a list item applies to your research, read the appropriate section before selecting a response.

Materials & experimental systems

n/a	Involved in the study	
☐	☒	Antibodies
☐	☒	Eukaryotic cell lines
☒	☐	Palaeontology
☒	☐	Animals and other organisms
☒	☐	Human research participants
☒	☐	Clinical data

Methods

n/a	Involved in the study	
☒	☐	ChIP-seq
☒	☐	Flow cytometry
☒	☐	MRI-based neuroimaging

Antibodies

Antibodies used	Anti-TccC3HVR antibody: custom-made anti-TccC3HVR rabbit polyclonal antibody (Cambridge Research Biochemicals). Dilution: 1:1000.<br>Anti-Cdc42 antibody: rabbit polyclonal antibody (Cell Signaling Technology, Cat. No. 2462). Dilution: 1:1000.<br>Anti-TEV antibody: rabbit polyclonal antibody (Novus Biologicals, Cat. No. NBP1-97669). Dilution: 1:500.<br>Secondary anti-rabbit antibody: HRP-conjugated goat anti-rabbit antibody (Biorad, Cat. No. 170-6515). Dilution: 1:2000.<br>Anti-pan-ADP-ribose binding reagent (Millipore, Cat. No. MABE1016). Dilution: 1:2000.
Validation	Anti-TccC3HVR antibody: validation for Western blot with purified TcdB2-TccC3 (0.5 - 3.0 pmol). Western Blot available upon request.<br>Anti-Cdc42 antibody: validated by the manufacturer according to the website (https://en.cellsignal.de/products/primary-antibodies/cdc42-antibody/2462).<br>Anti-TEV protease antibody: validated by the manufacturer according to the website (https://www.novusbio.com/products/tev-protease-antibody_nbp1-97669).<br>Anti-pan-ADP-ribose binding reagent: validated by the manufacturer according to the website (Millipore, Cat. No. MABE1016)

Eukaryotic cell lines

Policy information about cell lines

Cell line source(s)	HEK293T cells were obtained from ThermoFisher.

Authentication	Cell lines were not authenticated.
Mycoplasma contamination	Cells were not tested for Mycoplasma contamination.
Commonly misidentified lines (See ICLAC register)	n/a
